# COVID-19 Vaccination-Induced Cholangiopathy and Autoimmune Hepatitis: A Series of Two Cases

**DOI:** 10.7759/cureus.30304

**Published:** 2022-10-14

**Authors:** Mansoor Zafar, Katherine Gordon, Lucia Macken, Joe Parvin, Simon Heath, Max Whibley, Jeremy Tibble

**Affiliations:** 1 Gastroenterology/General Internal Medicine, Royal Sussex County Hospital, University Hospitals Sussex National Health Service (NHS) Foundation Trust, Brighton, GBR; 2 Gastroenterology, Royal Sussex County Hospital, University Hospitals Sussex National Health Service (NHS) Foundation Trust, Brighton, GBR; 3 Cellular Pathology, Royal Sussex County Hospital, University Hospitals Sussex National Health Service (NHS) Foundation Trust, Brighton, GBR; 4 Gastroenterology/Hepatology, Royal Sussex County Hospital, University Hospitals Sussex National Health Service (NHS) Foundation Trust, Brighton, GBR

**Keywords:** hematoxylin & eosin, magnetic resonance cholangiopancreatography (mrcp), c –reactive protein (crp), alt (alanine aminotransferase), emergency medical service, liver function tests (lfts), mrna

## Abstract

The coronavirus disease 2019 (COVID-19) pandemic has been associated with significant morbidity and mortality. Following the introduction of vaccines, various side effects have been reported. Whilst those reported may be attributed to the vaccine itself, at times, it may simply incite an immunological phenomenon. We present a case series of two patients who presented with symptoms of yellowing of the eyes and the skin along with fatigue, and tiredness, following vaccination for COVID-19. The diagnosis of post COVID-19-vaccination related hepatitis is one of the fewer, less understood, yet reported side effects associated with significant morbidity. The diagnosis of COVID-19 vaccination-related cholangitis is an outcome reported here for the first time to the best of our knowledge. It was alarming that both patients did not have any significant past history of medical ailments. A prompt assessment followed by investigations including liver biopsy assisted in a timely understanding of the phenomenon with complete resolution of the symptoms.

## Introduction

The phenomenon of the coronavirus disease 2019 (COVID-19) pandemic, to put simply, has struck the world with ‘shock and awe'. Severe acute respiratory syndrome coronavirus 2 (SARS-CoV-2) infection that was initiated in December of 2019 emerged from Wuhan, China, and to date remains a global pandemic [1]. A multitude of research papers and case reports were published. Many were focused on associations or demystifying the myths including low lymphocyte count [2], lower vitamin D levels [3], and association with high HbA1c levels [4] and suggesting the role of comorbidity towards association with morbidity and mortality [1-4].

Associations with diarrhea and inflammatory bowel disease were also written [5]. While the world tried to take a grasp of the pandemic through simple measures including isolation, ensuring face masks, and hand washing, there was an enormous surge in the pharmaceutical industry to develop vaccines as prevention and not just depend on herd immunity.

Soon the media outlets were talking about the groundbreaking technology of using messenger ribonucleic acid (mRNA) that implied injected vaccine coding at the cellular level to block the attachment of SARS-CoV-2 and hence an attempt to prevent sickness. This happened to be a brand-new rapid approach with claims for 95% effectiveness that was rapidly available to masses across the globe. This brand-new approach was also superior to the previous traditional vaccines that would take time for scientists to grow them into less mutagenic strains and/or non-mutagenic forms to help with acquiring immunity [6].

This led to the supply of various vaccines by providers including Pfizer, AstraZeneca, Moderna, and many others. These vaccines were made and distributed globally to curtail the rising incidence and prevalence of COVID-19 infection. The focus of the governments was to provide quickly for the masses in an attempt to overcome the huge global financial burden arising due to isolation, significant morbidity, and mortality. Not surprisingly, although the vaccines, in general, were massively a big relief to the stress of the people globally, and indeed did help to prevent the spread and avoidance of significant morbidity and mortality, there was an emergence of side effects. These side effects ranged from the development of rashes [7], pneumonitis [8], myositis [8], pericarditis [8], and even cerebral venous sinus thrombosis [9]. Although they were rarer occurrences, they did cause concerns about the overall effectiveness versus side effects of the new COVID-19 vaccines.

Multiple cases have been reported on the recent association between COVID-19 vaccination and autoimmune hepatitis (AIH). These include post Pfizer-BioNTech COVID-19 [10], post Moderna-COVID-19 (mRNA-1273) [11-13], and post first dose of mRNA-1273 SARS-CoV-2 vaccines [14]. A relationship between autoimmune hepatitis and concurrent COVID-19 infection itself has also been published [15]. Researchers at Oxford University Hospitals analyzed the data for 1000 patients with chronic liver disease and COVID-19 infection between March 2020 and October 2020, of which 70 patients had autoimmune hepatitis (AIH). The study proved no risk of death due to the continued use of immunosuppressive medications in AIH even in the setting of the COVID-19 infection [16].

As the unprecedented COVID-19 pandemic led to mass vaccination, vaccine-induced side effects, including AIH, have been brought to the surface. Whilst phase 4 clinical trials for most, if not all, COVID-19 vaccines are ongoing, the phenomenon of post COVID-19-vaccination side effects continues to remain a topic for discussion.

We report two cases: 1) a case of post COVID-19 vaccine-induced cholangiopathy, and 2) a patient who developed post COVID-19 vaccine AIH. 

## Case presentation

Case one

A 51-year-old man with no significant past medical history presented to the Emergency Department (ED) with complaints of fatigue, general malaise, and painless jaundice. He was referred to the gastroenterology team for advice. Baseline observations were all within normal range, however, he was icteric. Abdominal examination was normal. Blood analysis confirmed deranged liver function test (LFT) results (Table 1).

**Table 1:** Deranged Liver function test (LFT) results at hepatology review

Parameter	Unit of measurement	reference	Test result
Bilirubin	umol/L	0-21	290
Alanine transaminase (ALT)	iu/L	0-41	88
Alkaline phosphatase (ALP)	iu/L	40-129	487
C-reactive protein (CRP)	mg/L	0-5	10

**Figure 1:**
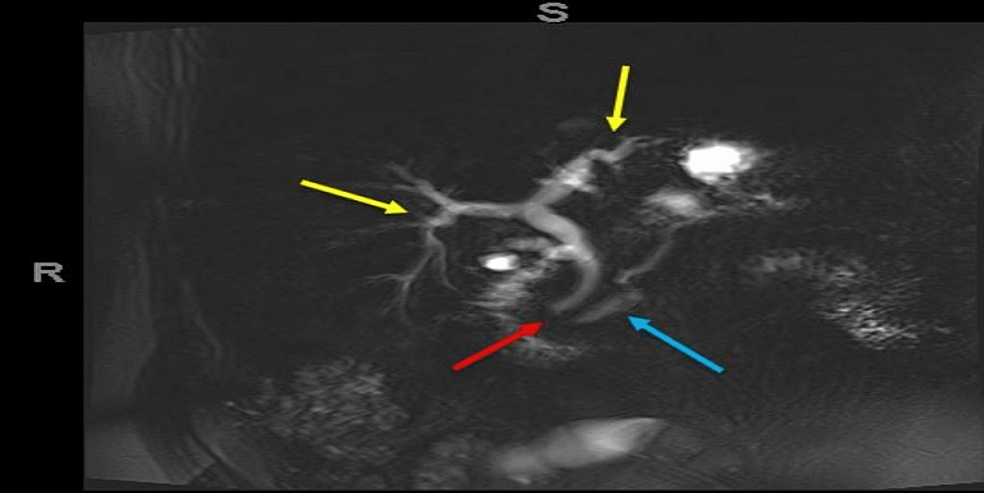
Magnetic resonance cholangiography (MRCP) diffusion-weighted image Bile duct stricturing (yellow arrows). Dilated proximal pancreatic duct (blue arrow) with prominent common bile duct (red arrow).

A full blood liver screen was also performed (autoimmune, inherited, and viral hepatitis causes along with immunoglobulin levels). A computed tomogram of the abdomen-pelvis (CT-AP) demonstrated biliary duct dilatation requiring magnetic resonance cholangiopancreatography (MRCP) for further evaluation, which showed bile duct stricturing and irregularity, raising the possibility of primary sclerosing cholangitis (Figure 1).

It was noticed that the patient had deranged LFT results done six months previously requested by the general practitioner (GP) when compared with the LFT results at the presentation (Table 2). 

**Table 2:** Comparison of deranged LFT results at presentation and six months ago * General Practitioner (Family Doctor); ** Alanine transaminase; ! Alkaline phosphatase; !! C-reactive protein

Parameter	Unit of measurement	reference	Test result at hepatology review	Test results 6 months ago, requested by GP^*^
Bilirubin	umol/L	0-21	290	157
ALT^**^	iu/L	0-41	88	141
ALP^!^	iu/L	40-129	487	362
CRP^!!^	mg/L	0-5	10	-

The patient explained that he had developed jaundice, fatigue, and tiredness which had lasted a week and settled, and hence he did not seek any further medical attention. He further disclosed that he had similar symptoms nine months prior, that only lasted three days, and similarly did not seek medical attention. He further recalled that each episode had occurred following the COVID-19 vaccination. Initially, he was vaccinated with AstraZeneca/Oxford COVID-19 (ChAdOx1 S {recombinant} nine months before (March 2021). Following this, he was given the same vaccine again three months later (June 2021), and finally, he took a booster (Pfizer-BioNTech) COVID-19 vaccine (January 2022). He explained the symptoms of jaundice occurred for a few days each time following the COVID-19 vaccination. Liver screen test results were negative. The estimated non-invasive, updated Roussel Uclaf Causality Assessment Method scale (RUCAM) was 1 (Table 3) [17]. 

**Table 3:** Updated RUCAM for the cholestatic or mixed liver injury of DILI and HILI RUCAM: Roussel Uclaf Causality Assessment Method scale; DILI: Drug-induced liver injury; HILI: Herb-induced liver injury (HILI) [17] ! A rise in ALP should be accompanied by raised levels for 5 prime nucleosidase (5’ nucleosidase) or γ-glutamyl-transpeptidase (GGT) in parallel with a hepatobiliary cause. ALP isotypes are helpful in divergent cases pointing to a bone or placental cause; !! pregnancy is a risk factor for cholestatic and mixed liver injury, and not for hepatocellular injury. * Group I (seven causes) HAV: Anti-HAV-IgM, Hepatobiliary sonography/color Doppler, HCV: Anti-HCV, HCV-RNA, HEV: Anti-HEV-IgM, anti-HEV-IgG, HEV-RNA, Hepatobiliary sonography/color Doppler sonography of liver vessels/endo-sonography/CT/MRC, Alcoholism (AST/ALT ≥ 2), Acute recent hypotension history (particularly if underlying heart disease) *Group II (five causes) Complications of underlying disease(s) such as sepsis, metastatic malignancy, autoimmune hepatitis, chronic hepatitis B or C, primary biliary cholangitis or sclerosing cholangitis, genetic liver diseases, Infection suggested by PCR and titer change for; CMV (anti-CMV-IgM, anti-CMV-IgG), EBV (anti-EBV-IgM, anti-EBV-IgG), HSV (anti-HSV-IgM, anti-HSV-IgG), VZV (anti-VZV-IgM, anti-VZV-IgG) **Patient's ALT 88 U/L, Upper limit of normal ALT 40 U/L, Patient's ALP 487 U/L, Upper limit of normal ALP 120 U/L, R-value 0.5, RUCAM score < 2. The cholestatic pattern of liver injury. Recommend imaging studies such as abdominal ultrasound) [18]

Items for Hepatocellular Injury	Updated RUCAM for the cholestatic or mixed liver injury of DILI and HILI.	Patient’s score- Case 1
1. Time to onset from the beginning of the drug/herb; 5–90 days (rechallenge: 1–90 days)	+ 2	+1
<5 or >90 days (rechallenge: >90 days)	+1	
Alternative: Time to onset from the cessation of the drug/herb (except for slowly metabolized chemicals: ≤30 days)	+1	
2. Course of ALP^!^ after cessation of the drug/herb (Percentage difference between ALP peak and upper level of normal); Decrease ≥ 50% within 180 days	+2	+1
Decrease < 50% within 180 days	+1	
No information, persistence, increase, or continued drug/herb use	0	
3. Risk factors Alcohol use (current drinks/day: >2 for women, >3 for men)	+1	+1
Alcohol use (current drinks/day: ≤2 for women, ≤3 for men)	0	
Pregnancy^!!^	+1	
Age ≥ 55 years	+1	
Age < 55 years	0	
4. Concomitant drug(s)/herb(s); None or no information	0	-3
Concomitant drug/herb with incompatible time to onset	0	
Concomitant drug/herb with compatible or suggestive time to onset	-1	
Concomitant drug/herb known as hepatotoxin and with compatible or suggestive time to onset delete marking right side above	-2	
Concomitant drug/herb with evidence for its role in this case (positive rechallenge or validated test)	-3	
5. Evaluation of groups I and II (for the alternative causes) *; All causes-groups I and II—reasonably ruled out	+2	
The 7 causes of group I ruled out	+1	
6 or 5 causes of the group I ruled out	0	
Less than 5 causes of group I ruled out	-2	
Alternative cause is highly probable	-3	
6. Previous hepatotoxicity of the drug/herb; Reaction labelled in the product characteristics	+2	0
Reaction published but unlabelled	+1	
Reaction unknown	0	
7. Response to unintentional re-exposure; Doubling of ALP with the drug/herb alone, provided ALT below 5 times the upper limit of normal before re-exposure	+3	+1
Doubling of ALP with the drug(s)/herb(s) already given at the time of the first reaction	+1	
Increase of ALP but less than the upper limit of normal in the same conditions as for the first administration	-2	
Other situations	0	
Total score for the case		1**

**Figure 2:**
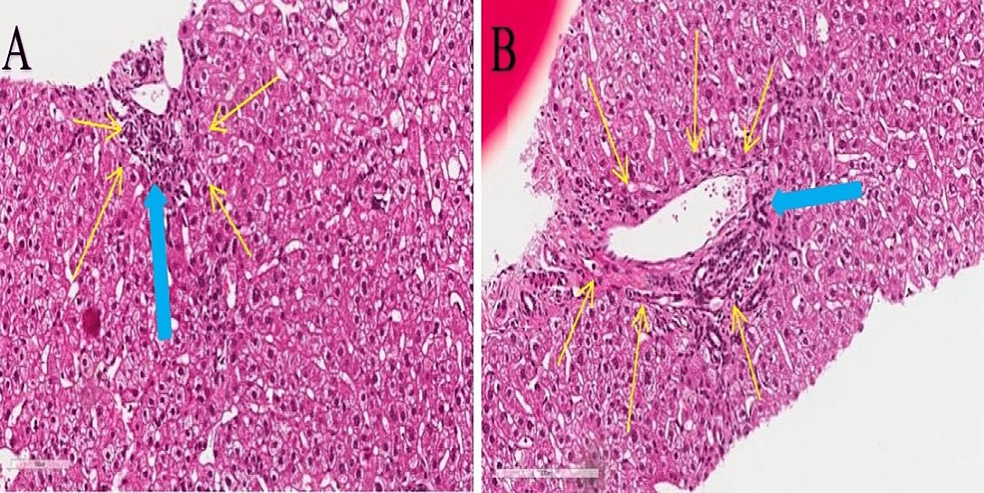
Liver tissue biopsy with H&E stain H&E: Haematoxylin & Eosin stain, Magnification: (A) x 100um, and (B) x 200um. Images show mild chronic portal inflammation (yellow arrows) with evidence of ductitis (blue bold arrow).

**Figure 3:**
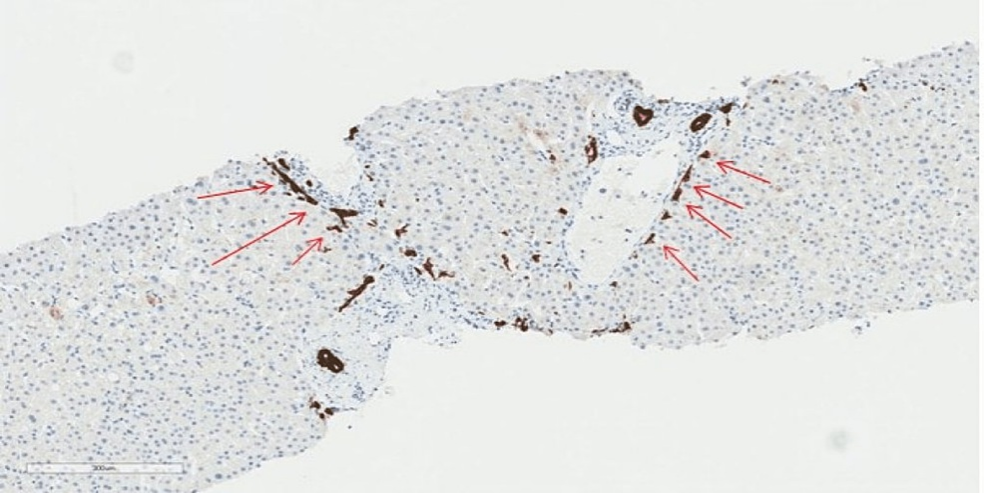
Liver tissue biopsy with CK7 stain CK7: Cytokeratin 7 stain with magnification x 300um. The image highlights a ductular proliferation at the interface (red arrows).

The patient agreed to undergo a liver biopsy which demonstrated features compatible with a drug-induced cholangiopathy (Figures 2, 3).

Subsequent liver function tests completely normalized with conservative treatment. The patient was advised to alternate COVID-19 vaccines for future boosters. Repeat LFTs at six months were normal.

Case two

**Figure 4:**
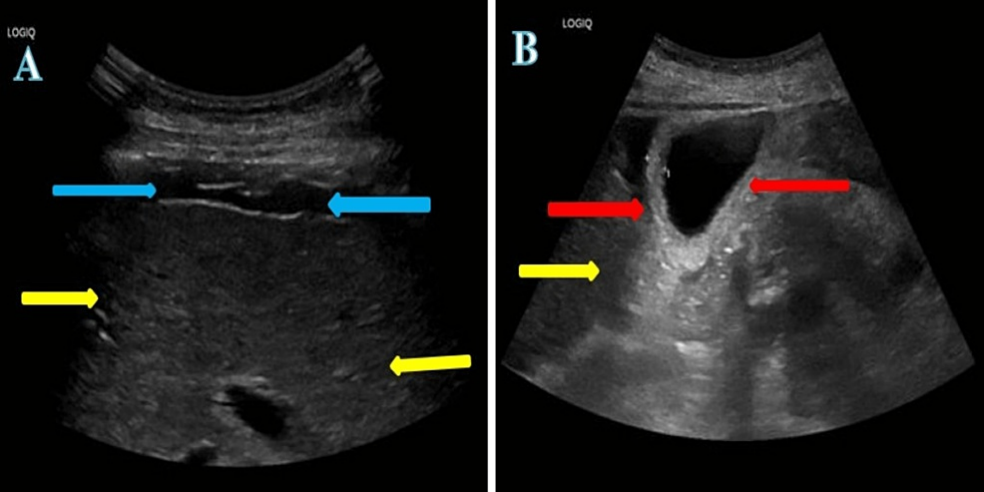
Ultrasound images A and B: ultrasound liver demonstrates mild irregular margin with coarse texture (yellow arrows) and markedly thickened gallbladder wall (red arrows), with mild to moderate ascites (blue arrows).

An 81-year-old lady presented with progressive jaundice for three weeks. On further inquiry, her stools had been slightly paler although she didn’t feel that her urine had been particularly dark and she reported mild intermittent pruritis. She did mention a somewhat scanty yellow vaginal discharge and two weeks ago was treated with a five-day course of nitrofurantoin for a urinary tract infection by the GP. She had a past history of depression for which she was on long-term citalopram. On examination, she was found to have normal baseline observations, afebrile but clearly icteric. Abdominal examination found mild hepatomegaly with no lymphadenopathy. There was no recent history of travel, and the patient only occasionally drank alcohol. Initial blood tests revealed significantly deranged LFTs (ALT 689 iu/L {0-33}, bilirubin 164 umol/L {0-21}, alkaline phosphatase 213 iu/L {35-104}, albumin 32 g/L {35-52}, total proteins 58 g/L {66-87} and globulins 22 g/L {18-36}). Full blood count showed haemoglobin 126 g/l (115- 165), mean corpuscular volume (MCV) 102.5 (83-101), ferritin 1405 ug/L (13-150), INR 1.2 (0.8-1.2), iron 36.8 umol/L (5.8-34.5), transferrin saturation 81% (25-45) and CRP 18 mg/L (0-5). Her urea/electrolytes/renal function was normal. Viral, metabolic, and hepatitis screens were normal. Serum immunoglobulin (Ig) levels showed IgG 13.8 g/L (7-16), IgA 2.9 g/L (0.7-4), IgM 0.9 g/L (0.4-2.3), Auto-antibody profile showed a negative screening of Anti-smooth muscle, Anti-mitochondrial, and Anti liver kidney microsomal antibodies but, a positive ANA (HEp2) with 1:320 titer. An abdominal ultrasound showed slightly coarsened echotexture with small volume ascites and some thickening of the gallbladder wall with no dilation of the common bile duct (Figure 4).

The patient stated that symptoms had started following her fourth COVID-19 vaccine (February 2022). An impression of possible post COVID-19 BNT162b2 mRNA (Pfizer-BioNTech) vaccine-induced hepatitis was made with a differential of autoimmune hepatitis initiated by COVID-19 vaccination. Since guidelines advise avoiding citalopram in patients with deranged LFTs, this patient was switched to sertraline. Genetic screening for hemochromatosis in view of raised ferritin and transferrin saturation was negative. The estimated non-invasive, updated RUCAM score was 6 (Table 4) [17].

**Table 4:** Updated RUCAM for the hepatocellular injury of DILI and HILI RUCAM: Roussel Uclaf Causality Assessment Method scale; DILI: Drug-induced liver injury; HILI: Herb-induced liver injury (HILI) [17] * Group I (7 causes) HAV: Anti-HAV-IgM, Hepatobiliary sonography/color Doppler, HCV: Anti-HCV, HCV-RNA, HEV: Anti-HEV-IgM, anti-HEV-IgG, HEV-RNA, Hepatobiliary sonography/color Doppler sonography of liver vessels/ endo-sonography/CT/MRC, Alcoholism (AST/ALT ≥ 2), Acute recent hypotension history (particularly if underlying heart disease), *Group II (5 causes) Complications of underlying disease(s) such as sepsis, metastatic malignancy, autoimmune hepatitis, chronic hepatitis B or C, primary biliary cholangitis or sclerosing cholangitis, genetic liver diseases, Infection suggested by PCR and titer change for; CMV (anti-CMV-IgM, anti-CMV-IgG), EBV (anti-EBV-IgM, anti-EBV-IgG), HSV (anti-HSV-IgM, anti-HSV-IgG), VZV (anti-VZV-IgM, anti-VZV-IgG). **Patient's ALT 689 U/L, Upper limit of normal ALT 40 U/L, Patient's ALP 213 U/L, Upper limit of normal ALP 120 U/L, R-value 9.7, RUCAM score > 5. The hepatocellular pattern of liver injury. Recommend acute viral hepatitis serologies, HCV RNA and autoimmune hepatitis serologies, and imaging studies such as abdominal ultrasound) [18].

Items for Hepatocellular Injury	Updated RUCAM score for the hepatocellular injury of DILI and HILI.	Patient’s score- Case 2
1. Time to onset from the beginning of the drug/herb; 5–90 days (rechallenge: 1–15 days)	+ 2	+2
<5 or >90 days (rechallenge: >15 days)	+1	
≤15 days (except for slowly metabolized chemicals: >15 days)	+1	
2. Course of ALT after cessation of the drug/herb (Percentage difference between ALT peak and upper limit of normal); Decrease ≥ 50% within 8 days	+3	+2
Decrease ≥ 50% within 30 days	+2	
No information or continued drug use	0	
Decrease ≥ 50% after the 30th day	0	
Decrease < 50% after the 30th day or recurrent increase	-2	
3. Risk factors Alcohol use (current drinks/day: >2 for women, >3 for men)	+1	+1
Alcohol use (current drinks/day: ≤2 for women, ≤3 for men)	0	
Age ≥ 55 years	+1	
Age < 55 years	0	
4. Concomitant drug(s)/herb(s); None or no information	0	-1
Concomitant drug/herb with incompatible time to onset	0	
Concomitant drug/herb with compatible or suggestive time to onset	-1	
Concomitant drug/herb known as hepatotoxin and with compatible or suggestive time to onset delete marking right side above	-2	
Concomitant drug/herb with evidence for its role in this case (positive rechallenge or validated test)	-3	
5. Evaluation of groups I and II (for the alternative causes) *; All causes-groups I and II—reasonably ruled out	+2	
The 7 causes of group I ruled out	+1	
6 or 5 causes of group I ruled out	0	
Less than 5 causes of group I ruled out	-2	
Alternative cause highly probable	-3	
6. Previous hepatotoxicity of the drug/herb; Reaction labelled in the product characteristics	+2	+1
Reaction published but unlabelled	+1	
Reaction unknown	0	
7. Response to unintentional re-exposure; Doubling of ALT with the drug/herb alone, provided ALT below 5N before re-exposure	+3	+1
Doubling of ALT with the drug(s)/herb(s) already given at the time of first reaction	+1	
Increase of ALT but less than N in the same conditions as for the first administration	-2	
Other situations	0	
Total score for the case		6**

**Figure 5:**
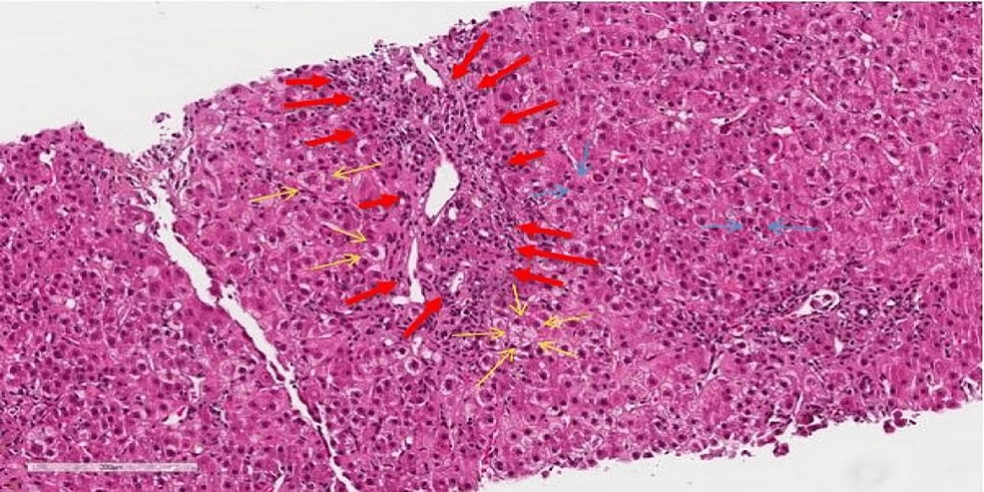
Liver tissue biopsy with H&E stain H&E: Haematoxylin & Eosin, Magnification x 200um The image shows mild chronic portal inflammation with scattered eosinophils (red bold arrows), Zone 1 with hepatocyte ballooning (yellow arrows), and lytic foci (blue arrows).

The patient underwent a liver biopsy that showed features compatible with a drug-induced liver injury (post COVID-19 vaccination) or autoimmune hepatitis secondary to drugs (Figure 5)*.*

She was started on spironolactone 50 mg once a day (OD) in respect of the ascites, prednisolone 40 mg OD with subsequent weaning by 5 mg every week, omeprazole 40 mg OD as stomach protection whilst on steroids, Adcal-D3® chewable tablets as bone protection whilst on steroids, and ursodeoxycholic acid 250 mg tablets three times a day (TDS) for pruritus/cholestasis. The patient’s LFTs started to normalize rapidly, however, towards the completion of the weaning regime of prednisolone (10 mg OD), her LFTs started worsening again with a rise in levels of bilirubin, alanine transaminase (ALT), and alkaline phosphatase (ALP) (Table 3)*.*

 

**Table 5:** Liver function tests over the three-month period. Source: Department of Biochemistry, Royal Sussex County, University Hospitals Sussex, Brighton. *OD; once a day; ! abnormal HFE variants associated with hemochromatosis include; C282Y and H63D; !!one of the normal variants of HFE in the general population not associated with hemochromatosis.

Parameters	units	Normal range	On presentation with the start of prednisolone 40 mg OD^*^	6 weeks post steroid (prednisolone 15 mg OD^*^)	7 weeks post steroid (prednisolone 10 mg OD^*^)	13 weeks (no steroids) azathioprine 75 mg OD^*^
bilirubin	umol/L	0-21	418	113	176	10
Alanine transaminase (ALT)	iu/L	0-33	689	297	205	34
Alkaline phosphatase (ALP)	iu/L	35-104	213	120	105	58
albumin	g/L	35-52	32	30	38	44
International Normalisation Ratio (INR)	-	0.8-1.2	1.2	1.2	1.1	1.1
iron	umol/L	5.8-34.5	36.8	-	-	-
Transferrin saturation	%	25-45	81	-	-	-
Ferritin	ug/L	13-150	1405	-	-	-
HFE gene^!^	-	-	c.187c g (p.his63asp)^!!^	-	-	-

She had a blood test for thiopurine methyltransferase (TPMT) levels and an increase in prednisolone to 20 mg with further weaning and commencement of azathioprine 10 mg daily subsequently normalized her LFTs which have remained normal off steroids.

## Discussion

The three commonly prescribed vaccines for prevention of the COVID-19 pandemic in the western hemisphere include Pfizer/BioNTech BNT162b2 mRNA, AstraZeneca/University of Oxford ChAdOx1-nCoV-19 chimpanzee adenovirus vector vaccine, and Moderna mRNA-1273 vaccine. Although the patients with liver disease were included in the rapid trials prior to supply to the masses, the proportion sample size was universally small [16,19]. The exact incidence of COVID-19 vaccine-induced liver injury is not available. However, the incidence of drug-induced injury to the liver every year has been reported to vary between 13.9 and 19.1 cases per 100,000 people. drug-induced liver injury incidence varies between 13.9 and 19.1 cases per 100,000 people [20-22].

In drug-induced liver injury, the pattern of injury to the hepatocytes with an offending drug has been varying between hepatitis with an increase in ALT, or cholestatic with an increase in the ALP. The third variant remains a mixed pattern associated with a simultaneous rise in levels of both ALT and ALP [23-25]. Most patients improve following stopping the medication with gradual resolution of symptoms and normalization of the laboratory liver function test results [25].

The updated RUCAM scale is a widely used non-invasive way to estimate the probability of drug-induced liver injury (DILI) and herb-induced liver injury (HILI). It implies analyzing serum levels of ALT, ALP, the upper limit of normal values (ULN), and the ratio (R) of elevation of baseline ALT to baseline ALP to predict the pattern of liver injury (Table 6) [18]. 

**Table 6:** Laboratory parameters and use of updated RUCAM scale towards estimating the pattern of liver injury RUCAM, Roussel Uclaf Causality Assessment Method; Reference no. [18] *a rise in ALP should be accompanied by raised levels for 5 prime nucleosidase (5’ nucleosidase) or γ-glutamyl-transpeptidase (GGT) in parallel with a hepatobiliary cause. ALP isotypes are helpful in divergent cases pointing to a bone or placental cause. **pregnancy is a risk factor for cholestatic and mixed liver injury, and not for hepatocellular injury ULN, upper limit of normal; ALT, alanine transaminase; ALP, alkaline phosphatase; >, more than; < less than; ≤, less than or equal to; R, ratio of elevation of baseline ALT to baseline alkaline phosphatase

Laboratory parameters of liver injury	ratio (R) of elevation of baseline ALT to baseline alkaline phosphatase (ALT/ULN)/(ALP/ULN)	Pattern of liver injury
ALP* > 2 ULN and ALT ≤ ULN, or if both ALT and ALP* are elevated.	R ≤ 2	Cholestatic**
ALT > 5 ULN and ALP* ≤ ULN, or if both ALT and ALP* are elevated.	R ≥ 5	hepatocellular
ALT > 5 ULN and ALP* > ULN	R > 2	mixed**

The variability in the probability based on the overall score can vary from highly probable to excluded variant (Table 7) [17].

**Table 7:** Probability of severity of liver injury with updated RUCAM scale RUCAM, Roussel Uclaf Causality Assessment Method scale; Reference no. [17] *a rise in ALP should be accompanied by raised levels for 5 prime nucleosidase (5’ nucleosidase) or γ-glutamyl-transpeptidase (GGT) in parallel with a hepatobiliary cause. ALP isotypes are helpful in divergent cases pointing to a bone or placental cause. **pregnancy is the risk factor for cholestatic and mixed liver injury, and not for hepatocellular injury. ULN, upper limit of normal; ALT, alanine transaminase; ALP, alkaline phosphatase; >, more than; < less than; ≤, less than or equal to; R, ratio of elevation of baseline ALT to baseline alkaline phosphatase

Category	Score	Likelihood (%)	Description
Highly probable	>8	>75	Highly probable, including “highly likely” and “definite.” The evidence for the drug causing the injury is beyond a reasonable doubt, clear, and convincing.
Probable	6–8	50–74	The preponderance of the evidence supports the link between the drug and liver injury.
Possible	3–5	25–49	The evidence for the drug causing the injury is equivocal but present.
Unlikely	1–3	<25	There is evidence that an etiological factor other than a drug caused the injury.
Excluded	<1	0	Causes could be excluded.

Mann et al. have published a case of cholangitis in a patient post COVID-19-vaccination, that resolved a few days following with a conservative approach [26]. However, in selected cases, it would become necessary to do a liver biopsy [24]. Camacho-Domínguez et al. have published a case report of a 79 years of age male who developed COVID-19 vaccine-induced auto-immune hepatitis presenting with deranged LFTs [27]. Although it does mention infiltrates including eosinophils, plasma cells, and lymphocytes, it does not mention interface hepatitis [27]. This phenomenon of Covid-19 vaccine-induced hepatitis has been reported in many studies [27-30].

A low antinuclear antibody (ANA) titer (e.g. 1:40, 1:80, or even 1:160), is often not associated with autoimmune disease association as compared with a high ANA titer (e.g. 1:640, 1:1280, or 1:2560) which has a much stronger association with disease presentation. An intermediate level as for our patient (e.g., 1:320) is less clear and in the context of clinical presentation an indication for further investigations such as a liver biopsy [31].

A recent mention of AIH post-BNT162b2 mRNA (Pfizer-BioNTech) COVID-19 vaccination has been published by Fimiano et al. [32] who reported a patient with AIH post-COVID-19 vaccination and suggested an increase in prevalence, and hence a need for increased awareness of possible COVID-19 vaccine-induced AIH [32].

Our case series may answer the query by Fimiano et al. [32] regarding a relapse in liver function following the completion of a weaning course of prednisolone. Our case series suggests that some patients may require longer-term immunosuppression with azathioprine, particularly in those presenting with autoimmune hepatitis as opposed to cholangiopathy. Increased awareness of the potential side effects of COVID-19 vaccination will hopefully enable early identification of such cases and instigation of treatment, however, this case series is not intended to raise concerns of the public toward the safety of COVID-19 vaccination.

These cases suggest a temporal causal link between COVID-19 vaccination and immune-related cholangiopathy and immune hepatitis. It can be difficult to ascertain the chronology of onset in relation to vaccinations and whether this could be perhaps subclinical. Definite direct causal links can be challenging to make. It would be interesting to see clinical studies aimed at identifying an association between various COVID-19 vaccines and the induction of autoimmune hepatitis and/or cholangiopathy.

## Conclusions

COVID-19 vaccine-related side effects have surfaced, affecting various organ systems. It is not our intention to reduce the uptake of such vital vaccines but to encourage an open discussion with patients about the potential side effects. A DILI such as we saw with these two patients is rare, however, it is important to be recognized as a potential side effect, and for patients to be encouraged to act upon any symptoms once they arise, even if it resolves quickly, as was the case with the first gentleman's jaundice. General practitioners (family doctors) also have a vital role in the periodic review, with appropriate blood tests in at-risk groups like those with a history of immune suppression, chronic liver disease, autoimmune disorders, diabetes mellitus, and genetic or inherited disorders in particular those with increased comorbidities.
